# A Treatise on Nervous Diseases

**Published:** 1886-07

**Authors:** 


A TREATISE ON NERVOUS DISEASES; THEIR SYMPTOMS AND TREATMENT. *A Text-book for Students and Practitioners, by* SAMUEL G. WEBBER, M. D., *Clinical Instructor in Nervous Diseases, Harvard Medical School; Visiting Physician for diseases of the Nervous System at the Boston City Hospital; Member of the Massachusetts Medical Society; Member of the American Neurological Association, etc., etc. Octavo, pp. ix, 415. New York:* D. APPLETON & COMPANY. *Chicago:* A. C. McCLURG & COMPANY.

"This book was commenced with the purpose of writing briefly, and including what is most essential for the study of nervous diseases within as small a compass as possible." So reads the preface, which further states that the work is not written for specialists, but for students and general practitioners, who (presumably) have little time to read.

It belongs to the class of works that are written down, so to speak, to the level of an inferior class of readers. The work is divided into four nearly equal divisions, dealing with the diseases of the brain, spinal-cord, peripheral and sympathetic nerves, and the unclassified or functional. A chapter is devoted to the consideration of each disease, preceded by a short bibliography. As to matters of fact, there are no serious misstatements but a faulty arrangement; a series of descriptions put together without any attempt at classification renders the work of little value to the novice, for whom it was evidently written. The fifteen illustrations are too few in number to be of great value in illustrating the text.

The work seems to have been compiled for the sake of writing a book; printed for the sake of publishing one; and may be bought by those who desire to own a "treatise" in a good binding and of fair typographical appearance.

We are still of the opinion that the best work for the beginner in neurology is a work written for specialists and used by them.

